# Study of prevalence and risk factors of chemotherapy-induced mucositis in gastrointestinal cancer using machine learning models

**DOI:** 10.3389/fonc.2023.1138992

**Published:** 2023-09-28

**Authors:** Lin Huang, Xianhui Ye, Fengqing Wu, Xiuyun Wang, Meng Qiu

**Affiliations:** ^1^ Division of Medical Oncology, Cancer Center, West China Hospital of Sichuan University, Chengdu, Sichuan, China; ^2^ Division of Medical Oncology, Colorectal Cancer Center, West China Hospital of Sichuan University, Chengdu, Sichuan, China; ^3^ Department of Abdominal Cancer, Cancer Center, West China Hospital of Sichuan University, Chengdu, Sichuan, China

**Keywords:** chemotherapy side effects, chemotherapy toxicity, cancer treatment toxicity, chemotherapy tolerability, gastrointestinal cancer, chemotherapy-induced mucositis, incidence, risk factors

## Abstract

**Objective:**

Chemotherapy-induced mucositis (CIM) significantly impacts clinical outcomes and diminishes the quality of life in patients with gastrointestinal cancer. This study aims to prospectively determine the incidence, severity, and underlying risk factors associated with CIM in this patient population.

**Methods:**

To achieve this objective, we introduce a novel Machine Learning-based Toxicity Prediction Model (ML-TPM) designed to analyze the risk factors contributing to CIM development in gastrointestinal cancer patients. Within the winter season spanning from December 15th, 2018 to January 14th, 2019, we conducted in-person interviews with patients undergoing chemotherapy for gastrointestinal cancer. These interviews encompassed comprehensive questionnaires pertaining to patient demographics, CIM incidence, severity, and any supplementary prophylactic measures employed.

**Results:**

The study encompassed a cohort of 447 participating patients who provided complete questionnaire responses (100%). Of these, 328 patients (73.4%) reported experiencing CIM during the course of their treatment. Notably, CIM-induced complications led to treatment discontinuation in 14 patients (3%). The most frequently encountered CIM symptoms were diarrhea (41.6%), followed by nausea (37.8%), vomiting (25.1%), abdominal pain (21%), gastritis (10.5%), and oral pain (10.3%). Supplementary prophylaxis was administered to approximately 62% of the patients. The analysis revealed significant correlations between the overall incidence of CIM and gender (*p*=0.015), number of chemotherapy cycles exceeding one (*p*=0.039), utilization of platinum-based regimens (p=0.039), and administration of irinotecan (*p*=0.003). Specifically, the incidence of diarrhea exhibited positive correlations with prior surgical history (*p*=0.037), irinotecan treatment (*p*=0.021), and probiotics usage (*p*=0.035). Conversely, diarrhea incidence demonstrated an adverse correlation with platinum-based treatment (*p*=0.026).

**Conclusion:**

In conclusion, this study demonstrates the successful implementation of the ML-TPM model for automating toxicity prediction with accuracy comparable to conventional physical analyses. Our findings provide valuable insights into the identification of CIM risk factors among gastrointestinal cancer patients undergoing chemotherapy. Furthermore, the results underscore the potential of machine learning in enhancing our understanding of chemotherapy-induced mucositis and advancing personalized patient care strategies.

## Introduction

Gastrointestinal (GI) cancer stands as a significant contributor to cancer-related morbidity and mortality on a global scale. Conventional chemotherapy has been recommended for patients exhibiting poor prognostic indicators, aiming to mitigate the risk of tumor recurrence and progression (1). Among the challenges posed by such therapies, mucositis emerges as a prevalent concern, characterized by ulceration and erythema of the mucosal lining within the GI tract. This affliction manifests in approximately 20-40% of patients undergoing traditional chemotherapy regimens (2, 3). Distinctively, oral mucositis is typified by ulceration and erythema affecting the oral mucosa, while GI mucositis commonly presents with symptoms encompassing pain, vomiting, nausea, and diarrhea. Given the widespread usage of cytotoxic treatments in GI cancer cases, an increased in the trajectory in the occurrence of chemotherapy-associated mucositis (CIM) has given rise to a pressing clinical issue (4). This issue brings significance changes specially detrimental impact on patients’ quality of life, treatment adherence, prolonged hospital stays, and overall clinical outcomes (5, 6).

Certain cytotoxic drugs such as platinum, irinotecan, and fluorouracil frequently cause CIM when FOLFOX, FOLFIRI, and S-1 are recommended during proper management of GI malignancies. Due to tumor occupation or surgical damage, CIM in GI cancer patients would aggravate the damage of the patient’s GI function and impaired prognosis compared with non-digestive tumors such as head and neck, and lung cancer. In previous studies that were focused on wild-type tumors, higher incidence rates of oral mucositis and diarrhea were observed (7–9). Some of the previously published studies focused on individual components of CIM revealed that the incidence and severity rate varied from patient to patient and those were regime-related. Potential risk factors observed such as gender, age, past medical history, as well as the type of drug used, its dosage, schedule, and administration route (10–12). However, even Multinational Association Of Supportive Care In Cancer and International Society of Oral Oncology (MASCC/ISOO) (2) and European Society for Medical Oncology (ESMO) clinical practice guidelines (13) for the management of oral and GI mucosa injury have proposed limited measurements mainly on the head and neck radiation-related and hematopoietic stem cell transplantation related mucositis.

Until now, the treatment for CIM consists of diverse drugs, not only having no established standard schedule but presenting inconsistent outcomes. Glutamine, probiotics, and a comprehensive elemental diet are widely applied as countermeasures for CIM (14). However, even those prescribed drugs should be pre-arranged before chemotherapy. Moreover, these drugs are usually administered unless severe complications occur and may themselves transiently bring about adverse effects, including pruritus, rash, erythema, tongue and mouth disorders, and taste alteration (15).

In recent years, machine learning (ML) and artificial intelligence (AI) have been used to forecast chemotherapy-related complications (16, 17). Owing to the early adoption of electronic chemotherapy recommendations, a rich source of past patient information regarding chemotherapy and gastrointestinal cancer and a subset of key parameters has been established. Novel data mining techniques incorporating ML methods can be utilized to analyze these data to produce more accurate, personalized predictions of the risk of gastrointestinal cancer. Machine learning can forecast the recurrence of gastric cancer patients after an operation.

There are very few studies focusing on the occurrence of CIM in GI cancers. Therefore, the current cross-sectional study is designed to obtain the overall incidence and severity of CIM in patients with gastrointestinal cancer. In addition, clinical features covering patient characteristics, inducements, and therapeutic factors of high-risk populations have also been examined. Hence, in this study, ML-based Toxicity Prediction Model (ML-TPM) has been proposed for analyzing the risk factors of CIM in GI cancer.

## Materials and methods

### Patients

This study enrolled consecutive in-hospital patients with gastric or colorectal cancer who had received at least one cycle of chemotherapy in the cancer center in our Hospital from Dec. 2018 to Jan. 2019. The Institutional Ethics Committee of the Hospital approved the protocol. The prior consent of the participants was taken before participation in the study. The exclusion criteria implied was: Patients with an ECOG score >2 had vomiting, diarrhea, or gastrointestinal bleeding before chemotherapy, with uncontrolled thyroid, diabetes, kidney or liver disorder. All included patients were interviewed via questionnaires which were face-to-face and recorded by two well-trained study nurses about (1) Personal information including age, gender, height, weight, and ECOG score; (2) Previous disease history including surgery or radiation history, other medical and medication history; (3) Disease info included tumor location, Tumor, Node, Metastasis (TNM) stage, chemotherapeutic regimens, and cycles were traced through electronic medical records system; (4) Symptomatic info covered the onset, duration, extent, and management of CIM like nausea and vomiting, diarrhea, abdominal pain, oral pain, and gastritis.

### Chemotherapeutic regimens and CIM management

In this study, we selected chemotherapeutic regimens by treating physicians according to clinical practice guidelines FOLFIRI regimen (irinotecan 150 mg/m^2^ i.v. on day 1, leucovorin at a dose of 200 mg/m^2^ i.v. on day 1, followed by bolus 5-FU400 mg/m^2^, and a 46 h infusion of 5-FU (2400 mg/m^2^) on days 1 to 2 were administered every 2 weeks) or mFOLFOX6 regimen (oxaliplatin 85 mg/m^2^ i.v. on day 1, leucovorin 100 mg/m^2^ i.v. on days 1 & 2 trailed by bolus 5-FU 400 mg/m^2^, and a 46-h infusion of 5-FU 2400 mg/m^2^ on days 1 to 2 were administered every 2 weeks) and XELOX regimen (Xeloda 2000 mg/m^2^/d for 1-14 days. A 2 h infusion of oxaliplatin (130 mg/m^2^) i.v. Day 1 for every 3 weeks was utilized for patients with colorectal cancer. SOX regimen (S1 80 mg/m^2^ on days 1 to 14 po. bid. and a 2 h infusion of oxaliplatin (130 mg/m^2^) i.v. on day 1 every 3 weeks) or XELOX regimen was used for gastric cancer. CIM management, including glutamine, probiotics, enteral nutrition, digestive enzymes, and Chinese herbs, was took by the patients.

### Endpoints and statistical analysis

In the current study, the first endpoint was the incidence of CIM at any grade of targeted CIM symptoms, including oral pain, abdominal pain, gastritis, nausea, vomiting, and diarrhea. Grades of CIM were scaled consistently with the National Cancer Institute Common Terminology Criteria for Adverse Event v4.0 (NCI-CTCAE v4.0). Secondary endpoints include correlation factors for CIM.

Both primary and secondary endpoints were analyzed as described previously (18). The accuracy of the TPM model was in ML-TPM setting was assessed as described previously (19).

### Statistical analysis

Data were set as ordered variables and were entered into a computerized database (SPSS statistical software, SPSS Inc., Chicago, IL). Kendall test was applied to detect the correlation between the incidence of CIM and patient demographics, including baseline information, chemo-regimens, and supplementary CIM prophylaxis. Statistical implication was accepted at the *p <*0.05 levels.

## Results

### Patients

A total of 447 patients with gastric (31.5%) or colorectal cancer (68.5%) who were scheduled for chemotherapy were timely interviewed between Dec. 2018 and Jan. 2019, and 100% valid questionnaires were collected and valid. The baseline characteristics have also been tabulated (**Table 1**). The middle age observed was 56 yrs. The mainstream of patients had normal nutritional status with a normal BMI score (74.9%) and lower ECOG score (ECOG=0, 85.7%; ECOG=1,13.6%), and the number of patients with stage IV disease was relatively high (48.3%). In previous treatment history, 329 of 447 patients (73.6%) had surgery, 61 patients (13.6%) received target therapy, and 89 patients (19.9%) had a history of radiotherapy. Large majority of the patients (62%, n=277), out of 447 patients received supplementary drugs (**Table 2**).

**Table 1:** Baseline characteristics.

Variable	N,(%)
Age,median,years	56
<70	399(89.3)
≥70	48(10.7)
Gender	
Male	278(63.2)
Female	169(37.8)
BMI^a^	
<18	37(8.3)
18~22.9	335(74.9)
23~24.9	66(14.8)
≥25	9(2)
ECOG^b^	
0	383(85.7)
1	61(13.6)
2	3(0.7)
Diagnosis	
Gastric cancer	141(31.5)
Colorectal cancer	306(68.5)
Tumor stage	
Stage I	7(1.6)
Stage II	47(10.5)
Stage III	160(35.8)
Stage IV	216(48.3)
Unknown	17(3.8)
Chemotherapy regimens	
Platinum Based	358(80.1)
Irinotecan Based	63(14.1)
Fu-i.v. Based	156(34.9)
Fu-oral Based	274(61.3)
Cycles	
1 cycle	91(20.4)
>1 cycle	356(79.6)
Previous treatment	
Operation	329(73.6)
Target agent	
Bevacizumab	47(10.5)
Cetuximab	12(2.7)
Radiotherapy	89(19.9)
Chinese herbs	147(32.9)

a BMI, Body Mass Index.

b ECOG, Eastern Cooperative Oncology Group.

**Table 2:** Proportion of supplementary prophylaxis.

Supplementary prophylaxis	N(%)
Enteral nutrition	157(35.1)
Probiotics	99(22.1)
Glutamine	21(4.7)
Digestive enzyme	34(7.6)
Vitamines	62(13.9)
Chinese herbs	147(32.9)

### Incidence rate and severity of CIM

In this study, 119 patients (26.6%) did not display any CIM symptoms during the chemotherapy courses. 328 patients (73.4%) suffered from CIM (**Table 3**), and 14 patients (3.1%) had to discontinue treatment as a result of intolerable CIM. The highest overall incidence of CIM was diarrhea (41.6%), nausea (37.8%), vomiting (25.1%), abdominal pain (21%), gastritis (10.5%), and oral pain (10.3%). Severe CIM (grade ≥3) happened in 4 patients (0.9%) with oral pain, 3 patients (0.6%) with abdominal pain (2 with grade 4 malaise), 2 patients (0.4%) with nausea, 3 patients (0.7%) with vomiting, 16 patients (3.6%) with diarrhea (3 with grade 4 malaise), no patient presented severe gastritis.

**Table 3:** The incidence of chemotherapy-related mucositis.

	Incidence of CIM,n (%)				
	Grade 0	Grade 1	Grade 2	Grade 3	Grade 4
**Oral pain**	401(89.7)	37(8.3)	5(1.1)	4(0.9)	/
**Abdominal pain**	353(79)	73(16.3)	18(4)	1(0.2)	2(0.4)
**Gastritis**	400(89.5)	43(9.6)	4(0.9)	0	0
**Nausea**	278(62.2)	143(32)	24(5.4)	2(0.4)	/
**Vomiting**	335(74.9)	92(20.6)	17(3.8)	3(0.7)	0
**Diarrhea**	261( 58.4 )	141(31.5)	29(6.5)	13(2.9)	3(0.7)
	None	Any symptom of CIM			
**Total**	119(26.6)	328(73.4)			

### The correlation between CIM incidence and patients’ clinical characteristics

Being a highly concerning issue, the overall incidence of CIM was significantly correlated with gender (*p*=0.015), chemotherapy cycles (*p*=0.039), platinum-based (*p*=0.039), and irinotecan-based treatment (*p*=0.003). No correlation was detected between the overall incidence of CIM and the administration of oral fluorouracil. We further analyzed the association of clinical characteristics and incidence of each type of CIM. Firstly, we investigated the correlation between all kinds of CIM and patients’ baseline characters (**Table 4**). The incidence of oral pain was positively correlated with poor ECOG and chemotherapy cycles. The incidence of abdominal pain was positively interrelated with gender/female and higher TNM stage. In the incidence of gastritis, a significant positive correlation was revealed between higher ECOG scores and chemotherapy cycles. A negative correlation was revealed with tumor location/colorectal location and a history of surgery. The incidence of nausea and vomiting presented some statistical similarities; they positively correlated with female gender and chemotherapy cycles >1 and negatively with older age. The surgical history was revealed as a risk factor for diarrhea. Among all the baseline characters, chemotherapy cycles>1 and gender/female were detected as significantly correlated with the incidence of varied CIM types, indicating that patients with multiple lines of chemotherapy and female patients should draw more attention to the high risk of CIM.

**Table 4:** The relevance of baseline characteristics and chemotherapy-related mucositis.

	Overall		Oral pain		Abdominal pain		Gastritis		Nausea		Vomiting		Diarrhea	
	Correlation coefficient	P-value	Correlation coefficient	P-value	Correlation coefficient	P-value	Correlation coefficient	P-value	Correlation coefficient	P-value	Correlation coefficient	P-value	Correlation coefficient	P-value
**Age**	-0.07	0.103	0.046	0.232	-0.058	0.135	0.037	0.341	-0.13	0.001	-0.114	0.003	-0.031	0.453
**Gender**	0.115	0.015	0.041	0.384	0.107	0.021	0.041	0.384	0.118	0.011	0.137	0.003	0.007	0.886
**BMI**	-0.037	0.421	-0.01	0.798	-0.054	0.158	<0.0001	0.994	-0.041	0.282	-0.071	0.062	-0.044	0.323
**ECOG**	0.043	0.362	0.116	0.013	0.069	0.138	0.111	0.018	0.017	0.709	-0.066	0.157	0.071	0.116
**Tumor location**	-0.08	0.094	0.072	0.124	<0.0001	0.992	-0.126	0.007	0.077	0.095	-0.007	0.883	-0.05	0.271
**TNM stage**	0.078	0.089	0.056	0.227	0.122	0.008	0.015	0.748	0.053	0.253	-0.007	0.883	-0.036	0.408
**Chemotherapy cycle**	0.098	0.039	-0.151	0.001	0.05	0.28	0.101	0.032	0.128	0.006	0.112	0.016	-0.062	0.176
**Surgery**	0.024	0.561	-0.043	0.286	-0.077	0.06	-0.084	0.044	0.031	0.448	0.002	0.961	0.084	0.037
**Target agents**	-0.019	0.686	-0.016	0.728	-0.064	0.172	-0.031	0.51	-0.052	0.268	-0.056	0.228	0.003	0.953
**Radiotherapy**	-0.004	0.935	-0.025	0.6	-0.037	0.43	-0.043	0.363	0.085	0.073	0.074	0.12	0.036	0.341

**Figure 1:**
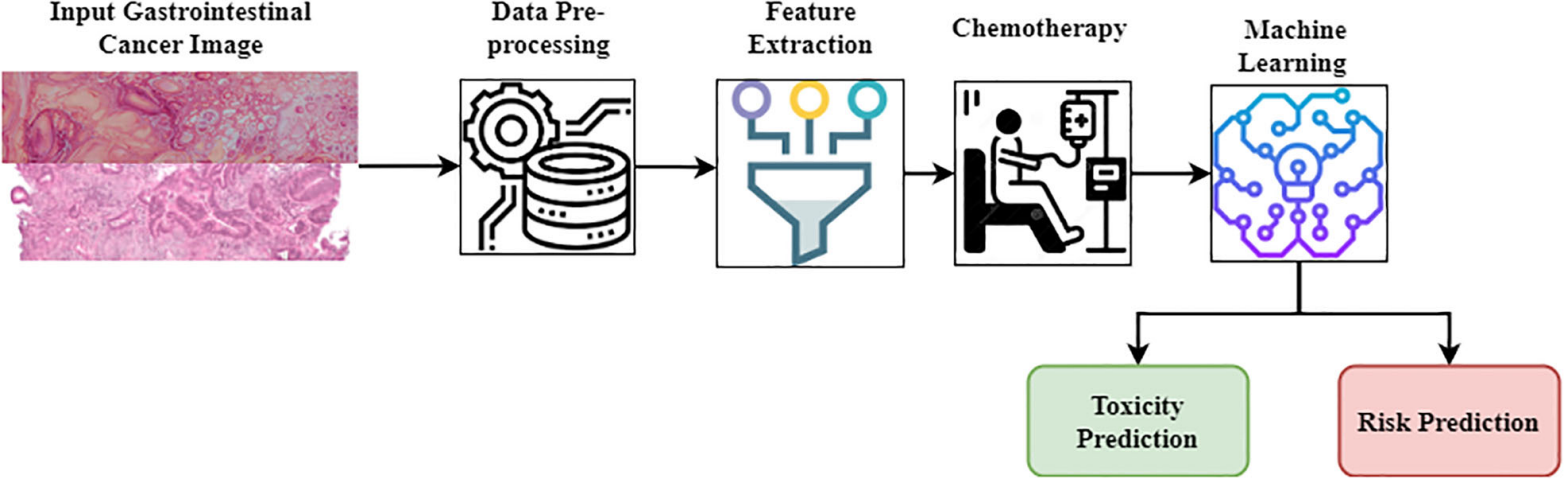
Proposed ML-TPM model. In the current study, the Kaggle dataset of gastrointestinal cancer images used to predict the prevalence and risk factors in clinical outcomes. Before deploying the ML model, it is essential to ensure the quality of the datasets using a preprocessing technique that includes feature extraction and standardization. Without any self-learning system, the feature extraction of a GI endoscopic image relies heavily on color and texture information. Chemotherapy (chemo) is the treatment of cancer using anti-cancer medications, either intravenously (through an IV line or central venous catheter) or orally (in the form of tablets). It is possible to treat cancer that has spread to other organs by using medications circulating throughout the body through circulation. Researchers are increasingly turning to machine learning to predict toxicity in gastrointestinal cancer due to the method’s speed, low cost, and high accuracy. A ML model is first developed to forecast toxicity after the chemical structure is represented using a computer-readable and interpretable technique. The primary treatment for gastrointestinal cancer is often systemic anti-cancer medications, with surgery, neo-adjuvant (chemotherapy), and postoperative adjuvants (chemotherapeutic) for high-risk improved stages (high-risk stage III and II).

**Figure 2:**
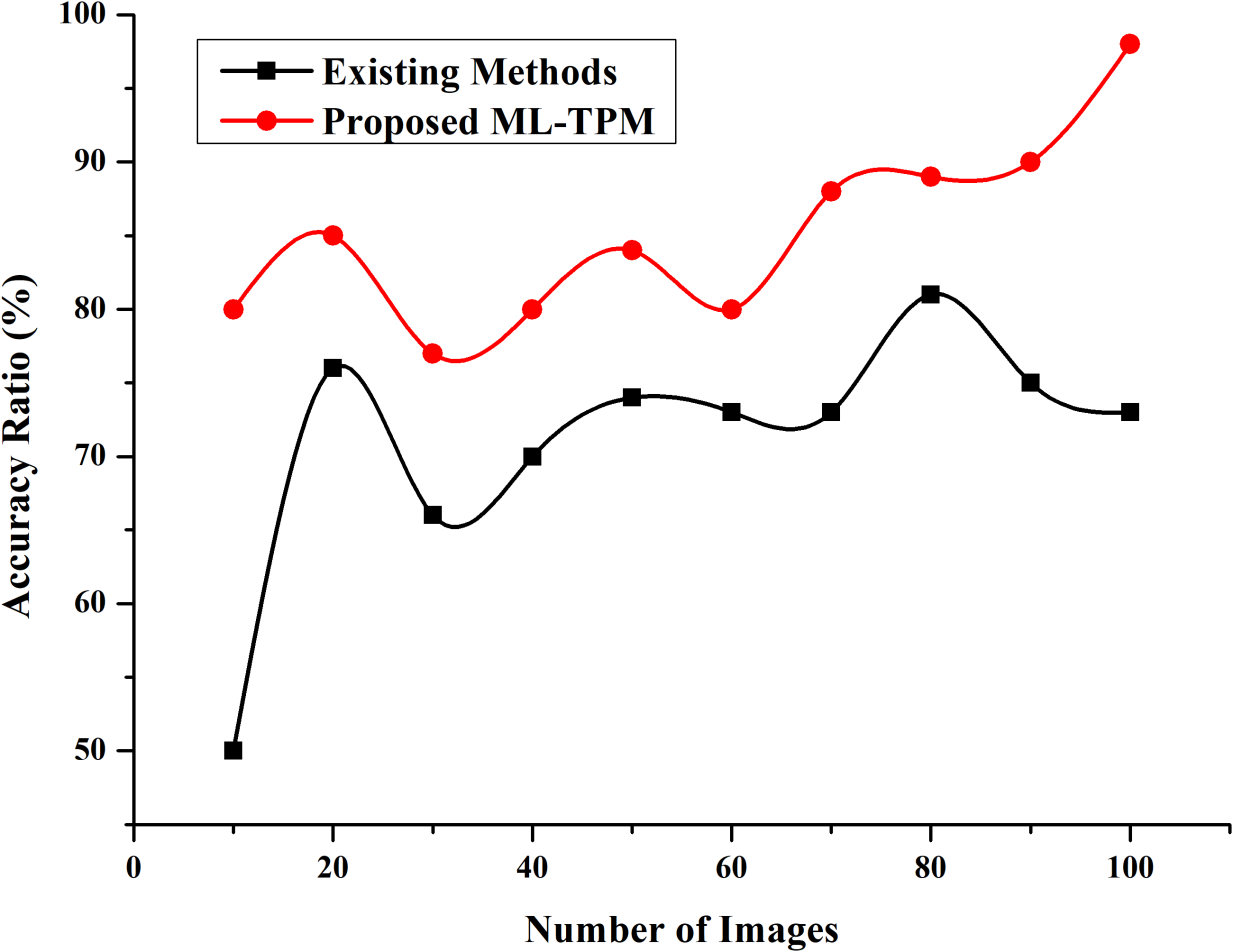
Accuracy Ratio. Developing a ML method that can automatically recognize lesion images from substantial GI cancer image datasets is necessary and meaningful to enhance detection efficiency and accuracy. The y-axis represent % accuracy ratio, and the x-axis represents the number of the images. The red colored line denotes proposed ML-TPM, and black line indicates existing methods.

Next, the incidence of subtypes of CIM varies with different chemotherapy regimens (**Table 5**). Platinum-based chemotherapy was positively linked with the incidence of abdominal pain and negatively linked with the incidence of diarrhea. Irinotecan-based chemotherapy was positively linked with the incidence of overall CIM and diarrhea, while negatively interrelated with the incidence of oral pain and abdominal pain; Fu-i.v. based chemotherapy was only positively correlated with oral pain; Fu-oral based treatment was negatively correlated with the incidence of oral pain and gastritis. The predictive model used, along with its accuracy, was depicted in **Figures 1**, **2**.

**Table 5:** The relevance of chemotherapy regimen and supplementary prophylaxis with chemotherapy-related mucositis.

Parameters	Overall		Oral pain		Abdominal pain		Gastritis		Nausea		Vomiting		Diarrhea	
	Correlation coefficient	*p*-value	Correlation coefficient	*p*-value	Correlation coefficient	*p*-value	Correlation coefficient	*p*-value	Correlation coefficient	*p*-value	Correlation coefficient	*p*-value	Correlation coefficient	*p*-value
**Platinum Based**	-0.098	0.039	0.051	0.275	0.113	0.015	0.086	0.068	0.021	0.643	0.002	0.966	-0.101	0.026
**Irinotecan Based**	0.142	0.003	-0.1	0.034	-0.143	0.002	-0.032	0.5	-0.033	0.48	-0.059	0.203	0.105	0.021
**Fu-i.v. Based**	0.016	0.731	0.154	0.001	0.019	0.682	-0.02	0.668	0.01	0.826	-0.024	0.605	-0.039	0.394
**Fu-oral** **based**	-0.032	0.502	-0.167	<0.0001	-0.044	0.34	-0.042	<0.0001	0.009	0.843	0.018	0.695	0.045	0.325
**Enteral nutrition**	0.019	0.687	-0.038	0.421	0.046	0.333	0.023	0.63	-0.023	0.63	-0.047	0.322	-0.051	0.285
**Probiotics**	0.06	0.202	0.054	0.256	-0.007	0.886	0.054	0.256	0.031	0.519	0.031	0.518	0.1	0.035
**Glutamine**	-0.01	0.836	-0.007	0.88	-0.011	0.82	-0.007	0.88	0.001	0.978	0.018	0.704	-0.016	0.738
**Digestive enzyme**	0.077	0.102	0.259	<0.0001	0.1	0.034	0.067	0.159	0.02	0.674	-0.03	0.532	0.083	0.079

### The correlation between CIM incidence and supplementary prophylaxis

In the current study, 277 (62%), out of 447 patients received supplementary drugs, including enteral nutrition, glutamine, probiotics, digestive enzymes, and Chinese herbs. Among them, the patients who choose enteral nutrition are the most, accounting for 35.1%. The impact of supplementary prophylaxis on the incidence of CIM was also recorded (**Table 5**). There was no statistically important correlation between the total incidence of CIM and the administration of supplemental elements. Current results were unexpected and demonstrated that oral pain was positively associated with a digestive enzyme and vitamin intake, and diarrhea incidence was positively associated with probiotics intake.

## Discussion

The present study reported a high incidence of CIM (73.4%) in gastric or colorectal cancer in Chinese patients receiving combination chemotherapy. However, severe CIM was rare (0.4-3.6%), and only 14 patients (3.1%) discontinued chemotherapy due to intolerable CIM; this fact mimics previous findings (11). Despite that low-grade CIM was dominant, they present as oral or gastrointestinal adverse effects that could impair patients’ quality of lives, dosage tolerance, and motivation for treatment and may ultimately result in worse survival.

Herein, it was found that clinical characteristics for CIM to identify high-risk GI cancer patients for preventing management. The overall incidence of CIM significantly positively correlated with gender and chemotherapy cycles; female and chemotherapy cycle>1 were highlighted as risk factors for CIM and diverse CIM types, and the findings remained consistent with the literature (20). Up to now, certain systematic reports on the vulnerability factors of CIM were published, most of which just focused on partial symptoms of it, such as oral mucositis (OM) and diarrhea, and tend to be reported unless severe conditions occurred. Few studies suggested that advanced age, a lack of craving, and the duration of chemotherapy might contribute to OM in breast, lung, and gastrointestinal tract cancer. At the same time, tumor-specific correlations are not mentioned (21). The history of chemotherapy and the number of chemotherapy cycles was regarded as involved in developing CIM (21, 22). Among the GI cancer population, we found patients who expected more than 1 cycle of chemo-therapeutic displayed a high incidence of overall CIM and oral pain, gastritis, nausea, and vomiting. Meanwhile, female patients presented a high risk of overall CIM, abdominal pain, nausea, and vomiting. Besides, younger patients showed a higher incidence of nausea and vomiting. Considering the similar trend in women, we suggest defining young females as a high-risk population for CIM. Tumor location, TNM stage, and surgery history had no significant correlation with the overall incidence of CIM but correlated with some malaise like gastritis and abdominal pain, respectively, which were recognized free of chemotherapy inducement but more relevant to disease or operation factors. Notably, a history of target agents and radiotherapy did not increase CIM incidence in our population, inconsistent with some articles which believe target and radiation therapy can lead to mucositis (13).

Based on our analysis, platinum and irinotecan significantly correlated with CIM incidence in opposite directions. Platinum-based treatments were negatively correlated with an overall incidence of CIM and diarrhea, while irinotecan-based treatment positively correlated with an overall incidence of CIM and diarrhea. 5-FU-based treatment was not correlated with the incidence of CIM, whether administered intravenously or orally. Among the chemotherapy agents recommended utilized for patients with GI cancer, oxaliplatin, irinotecan, and 5-FU are three drugs with the maximum risk for CIM, with their direct or indirect damage leading to the breakdown of the mucosal barrier, crypt cell death, and lastly, mucosal inflammations (11). Chemotherapy-induced diarrhea and OM are usually reported with 5-fu and irinotecan (8, 23), and nausea and vomiting are published more with irinotecan and oxaliplatin (24). The present study showed irinotecan had been related to multiple CIM sub-symptoms: diarrhea (positively), oral pain, and abdominal pain (negatively). Regarding the development of oral pain, 5-FU given through oral acted negatively rather than intravenous delivery. Thus, we would like to conclude that it’s necessary to pay more attention to CIM toxicities in the course of irinotecan involving chemotherapy and intravenous administrated of 5-FU.

In this cross-sectional study, an initial observation was that a high proportion (62%) of supplements includes glutamine, probiotics, enteral nutrition, digestive enzymes, and Chinese herbs in GI patients to alleviate or prevent CIM. However, we did not obtain a beneficial correlation between all mentioned supplementary drugs and the incidence of CIM or CIM sub-symptom. Due to insufficient evidence, management standards regarding CIM in gastrointestinal cancer are still inaccessible and inconsistent. A mass of articles discussed complementary agents for treating CIM. Glutamine could protect bowel mucosa from chemotherapy-induced DNA damage through the production of reactive oxygen species and apoptosis through the expression of inflammatory cytokines, such as tumor necrosis factor α (TNFα) and interleukin (IL)-1*β*, IL-6 (25, 26). Probiotics also counter the pathophysiology of mucositis with the effect of relieving dysbacteriosis caused by chemo-treatment. A recent meta-analysis study (27) has revealed that probiotics decreased the occurrence of diarrhea in cancer patients (95% CI 0.34-0.78 OR=0.52). Chinese herbs such as Rhodiolaalgida may possess anti-inflammation effects and quickly heal mucosa ulcers (28, 29). However, none of them raise high-quality evidence for their limitations in sample size or inadequate design. On the contrary, results in the present research failed to verify the relationship between supplementary prophylaxis and overall CIM incidence. At the same time, probiotics, digestive enzymes, and vitamins were significantly correlated with sub-types of CIM, despite non-beneficial effects. Considering varied types of supplementary drugs having many compounds, dosages, and pharmacodynamics, it is pretty hard to conduct further stratified analyses to discover potentially effective drugs. To determine the effect of supplementary prophylaxis on CIM, a prospective, multiple-center, randomized, controlled clinical trial for more valid evidence for preventing the management of CIM was crucial.

## Conclusion

This study was focused on application of the Machine Learning-based Toxicity Prediction Model (ML-TPM), designed to analyze the underlying risk factors associated with chemotherapy-induced mucositis (CIM) in GI cancer patients. Our cross-sectional assessment highlights a prevalent occurrence of CIM among patients undergoing chemotherapy for gastrointestinal cancer. Notably, female patients, those subjected to more than one chemotherapy cycle, and those treated with irinotecan or platinum-based regimens exhibit heightened susceptibility to CIM. Furthermore, we observe that a substantial portion (50%) of patients opt for supplementary prophylactic measures to manage CIM symptoms. It remains imperative to investigate the potential efficacy of supplementary prophylaxis, particularly for high-risk patients. Nevertheless, it’s important to acknowledge the limitations of this observational study, including its modest sample size, short-duration timeframe, and single-institute design. Additionally, variability and irregularity in the use of supplementary drugs for CIM by patients add complexity to the findings. To enhance the rigor of our findings, we recommend the execution of a prospective interventional study involving a larger and more diverse patient population. The noteworthy positive correlations established in this study were between CIM incidence, gender, and chemotherapy cycles in concordance with the previous study. In particular, our study underscores female gender and undergoing more than one chemotherapy cycle as significant risk factors for diverse CIM types. Looking ahead, our research paves the way for future investigations into biomarkers that could facilitate the optimization of precision treatment strategies for gastrointestinal cancer through computer-aided diagnostic tools. This pursuit holds the potential to contribute significantly to advancing personalized therapeutic approaches in this domain.

## Data availability statement

The original contributions presented in the study are included in the article/supplementary material. Further inquiries can be directed to the corresponding author.

## Ethics statement

The studies involving humans were approved by Ethics Committee of West China Hospital of Sichuan University. The studies were conducted in accordance with the local legislation and institutional requirements. The participants provided their written informed consent to participate in this study.

## Author contributions

Study conception and design: LH, XY. Data collection: FW, XW. Analysis and interpretation of results: LH, XY, MQ. Draft manuscript preparation: MQ. All authors reviewed the results and approved the final version of the manuscript.
